# Anti-fibrotic effects of valproic acid in experimental peritoneal fibrosis

**DOI:** 10.1371/journal.pone.0184302

**Published:** 2017-09-05

**Authors:** Elerson C. Costalonga, Luiza J. de Freitas, Deise da S. P. Aragone, Filipe M. O. Silva, Irene L. Noronha

**Affiliations:** 1 Laboratory of Cellular, Genetic, and Molecular Nephrology, Renal Division, University of São Paulo Medical School, São Paulo, Brazil; 2 Center for Cellular and Molecular Studies and Therapy (NETCEM), University of São Paulo Medical School, São Paulo, Brazil; Hospital Universitario de la Princesa, SPAIN

## Abstract

**Background:**

Progressive fibrous thickening of the peritoneal membrane is a complication of long-term peritoneal dialysis (PD). TGF-β/Smad pathway activation, inflammation, and neoangiogenesis play important roles in peritoneal membrane (PM) changes induced by PD. Recently, histone deacetilase inhibitors (HDACi) have shown anti-fibrotic and anti-inflammatory effects in different experimental models. These drugs prevent deacetylation of histones causing a loosen chromatin, which in turn induce the expression of some anti-fibrotic genes. In addition, acetylation may increase the activity of proteins involved in tissue fibrosis, such as Smad7. Here, we explored the effect of valproic acid (VPA), an HDACi, on the development of peritoneal fibrosis (PF) in rats.

**Methods:**

PF was induced by daily intraperitoneal injections of 0.1% chlorhexidine gluconate (CG) for 15 consecutive days. Male Wistar rats (250–300 g) were divided into 3 groups: CONTROL, control rats receiving only vehicle; PF, peritoneal fibrosis induced in rats; PF+VPA, rats with PF treated with VPA (300 mg/kg/day by gavage). PF was assessed by Masson’s trichrome staining. Inflammation and fibrosis-associated factors were assessed by immunohistochemistry, immunofluorescence, multiplex analysis, and qPCR.

**Results:**

Treatment with VPA significantly reduced PM thickness and the expression of myofibroblasts, besides preventing loss of ultrafiltration capacity of the PM. The upregulation of profibrotic factors (TGF-β, fibronectin, and Smad3) in the PF group was significantly ameliorated by VPA. VPA modulated the TGF/Smad pathway, inhibiting phosphorylated Smad3 expression and inducing an increased Smad7 expression in the FP+VPA group. The neoangiogenesis and the expression of proinflammatory cytokines (TNF-α, IL-1β, MCP-1) observed in the PF group was significantly reduced by VPA.

**Conclusions:**

Our results indicate that VPA suppressed experimental PF through modulation of the TGF-β/Smad pathway. Interestingly, VPA treatment induced a higher expression of antifibrotic factors, such as Smad7. These results suggest that VPA may represent a potential strategy for treating long term PD complications.

## Introduction

Despite the predominance of hemodialysis as renal replacement therapy, the best method of dialysis for patients with end-stage renal disease has not been established. Recent studies have indicated that the relative mortality risk of patients undergoing peritoneal dialysis (PD) may be lower compared to patients on hemodialysis, especially for the first two years on renal replacement therapy [1, 2]. However, the long term exposure to PD fluids, peritonitis, and genetic factors induce inflammation, neoangiogenesis, and fibrosis of the peritoneal membrane (PM) [3], which impair its function, leading to technique failure [3, 4]. Furthermore, some patients develop encapsulating peritoneal sclerosis (EPS), a rare condition of excessive peritoneal fibrosis with high mortality rates [5, 6]. To date, there has been no effective treatment available to prevent or stop these processes.

The pivotal role of the TGF-β/Smad signaling pathway in the pathogenesis of peritoneal changes induced by PD have been demonstrated [7–10]. TGF-β stimulates fibroblast proliferation, increases the production of extracellular matrix component deposition, and induces neoangiogenesis in the peritoneum [7]. Blocking TGF-β arrests peritoneal fibrosis (PF) in experimental studies [7]. The induction of profibrotic genes by TGF-β signal transduction is mediated by phosphorylation of receptor-regulated Smads (Smad2 and Smad3). On the other hand, inhibitory Smads (Smad6 and Smad7) are transcriptionally induced by TGF-β and negatively regulate these pathways, establishing an important negative feedback loop. Thus, inhibiting TGF-β or enhancing Smad7 expression likely represents an effective therapy for peritoneal fibrosis.

One important factor that regulates chromatin structure and consequently gene expression is the acetylation level of histones. This process is enzymatically controlled by histone acetyltransferases (HAT) and histone deacetylases (HDAC). The HDAC removes acetyl group from histones leading to a condensed and inactive chromatin [11]. Recently, HDAC inhibitors have shown antifibrotic effects in obstructive and adriamycin nephropathy models [12, 13]. These drugs prevent deacetylation of histones inducing an open chromatin which, in turn, may facilitate the expression of some antifibrotic genes such as Bone Morphogenic Protein-7 (BMP-7) [14]. In addition, acetylation may modulate the activity of proteins involved in tissue fibrosis[15]. For example, post-translational acetylation protects Smad7 against ubiquitination and degradation [16, 17]. Through histone modifications or by regulating the activity of non-histone proteins, evidence points to anti-inflammatory and antifibrotic effects of HDAC inhibitors [11, 18].

Valproic acid (VPA) is a short chain fatty acid clinically used as an anticonvulsant drug. Of note, VPA has been described as a histone deacetylase inhibitor (HDACi) with anti-inflammatory and antifibrotic actions [19]. By different mechanisms such as reducing macrophage (MΦ) infiltration, and attenuating the expression of TGF-β, VPA has been shown to inhibit fibrosis in liver [20], kidney [12], and heart [21] experimental models. Furthermore, VPA treatment reduced inflammatory cellular infiltration and expression of proinflammatory cytokines, preventing ischemic acute kidney injury in rats [22]. We therefore hypothesized that VPA could inhibit peritoneal fibrosis (PF). To investigate this possible effect, the effect of VPA was analyzed in rats submitted to an experimental model of PF induced by intraperitoneal (IP) chlorhexidine gluconate injections.

## Materials and methods

### Animals

The experiments were conducted using male *Wistar* rats, weighing from 250 g to 300 g, obtained from a colony at the University of São Paulo, Brazil. The animals were housed in rodent cages in a 22°C room with a 12-h light–dark cycle with free access to standard rat chow and water. All procedures were approved by the Ethical Research Board of the University of Sao Paulo, Brazil (Approval number: 145/14).

### Peritoneal fibrosis model and experimental groups

Peritoneal fibrosis was established by daily IP injection of 0.1% chlorhexidine gluconate (CG) in 15% ethanol dissolved in saline, as previously described [23]. Rats received injections of CG at a dose of 1 mL/100 g body weight into the peritoneal cavity for 15 consecutive days. Control rats were injected with an equal volume of 0.9% saline. To investigate the role of histone deacetylase blockade in peritoneal fibrosis, valproic acid (VPA; Depakene, Abbott, Illinois, USA) was administered daily at a dose of 300 mg/kg body weight for 15 days by *gavage*. The doses of VPA were selected based on previous studies [22, 24, 25].

After a run-in period of 15 days, 28 rats were assigned to 3 groups: **Control group**, normal rats, receiving only vehicle and IP saline injections from day 16 to 30 (n = 8); **Peritoneal Fibrosis (PF) group**, rats injected with CG for 15 consecutive days, from day 16 to 30 (n = 10); and **Peritoneal Fibrosis treated with Valproic Acid (PF+VPA) group**, PF rats treated with VPA by oral administration simultaneously for 15 days from day 16 to 30 (n = 10). To study the effect of VPA treatment on body weight, peritoneal thickness, and peritoneal function of normal rats, an additional group of normal animals treated with VPA **(Control + VPA)** was used (n = 5).

At day 30, euthanasia was performed by IP injection of sodium pentobarbital (100 mg/kg). Samples of the PM from the anterior abdominal wall away from the injection points were carefully dissected, immediately frozen in liquid nitrogen, and stored at– 70°C for polymerase chain reaction (PCR) and Multiplex analyses. Additional sections were fixed in Dubosq-Brazil solution for 45 minutes and then post-fixed in buffered 4% formaldehyde solution.

### Peritoneal membrane histomorphometric analysis

Peritoneal fibrosis was evaluated in sections (3 μm) stained with Masson’s Trichrome. At least 10 pictures at a 200x magnification were taken from each rat, and the thickness (μm) from all photomicrographs were measured. Then, a mean peritoneal thickness from each rat was calculated. For this procedure, we used digitized images and image analysis software (Image-Pro Plus Software 7.0, Media Cybernetics Inc., Bethesda, USA).

### Peritoneal function

A 2-hour peritoneal equilibrium test was performed at the end of the experiments. Each rat received IP injection of 0.09 ml/g of 4.25% peritoneal dialysis fluid, and 2 hours later rats were anesthetized. The abdominal cavity was opened along the linea alba and peritoneal fluid was collected with disposable sterile syringe under sterile conditions for biochemistry testing. The net ultrafiltration volume (UF) was determined by subtracting the drained volume from infused volume. The glucose concentration of the dialysate was determined and used to calculate the D_2_/D_0_ (the ratio of 2-hour to 0-hour glucose concentration of the dialysate) to assess the transport of small solutes [26]. The sodium gap (Na gap), determined by subtracting the sodium concentration of drained volume from infused PD fluid, was used as a surrogate marker of free water transport in the peritoneum [27].

### Immunohistochemistry

Immunohistochemical analysis was performed using paraffin-embedded sections by an indirect method, as previously described [28]. In brief, after deparaffinization and rehydration, antigenic recovery was obtained by heat in citrate buffer (pH 6.0). Endogenous peroxidase activity was then blocked and slides were incubated at 4°C overnight with the following monoclonal antibodies: anti-rat ED1 (macrophages; Serotec, Oxford, UK) diluted 1:200, anti-rat α-smooth muscle actin (α-SMA; Sigma Chemical Co., St. Louis, USA) diluted 1:400, and phosphorylated-Smad3 (pSmad3; Abcam, Cambridge, UK) diluted 1:1000. After incubation with the primary antibodies, the slides were submitted to a second reaction either with rat-adsorbed biotinylated anti-mouse IgG (Vector Labs, Burlingame, USA) or with biotinylated anti-rabbit IgG (Vector Labs, Burlingame, USA). To complete the sandwich, sections were incubated with streptavidin-biotin-alkaline phosphatase complex (Dako, Glostrup, Denmark). Negative control experiments were performed by omitting the incubation with the primary antibodies. Quantitative analysis of ED1 and pSmad3 positive cells present in the peritoneum was carried out in a blinded fashion under × 200 microscopic magnification, and expressed as cells/mm^2^. The α-SMA staining area (%) was calculated relative to the whole peritoneal area using Image-Pro Plus 7.0 software (Media Cybernetics, Inc., Bethesda, USA).

### Immunofluorescence

After deparaffinization and antigenic recovery by heat in citrate buffer (pH 6.0), the sections were permeabilized with Triton x-100 0.2% in phosphate buffered solution (PBS) for 5 minutes, followed by incubation with blocking solution of 5% BSA in PBS at room temperature for 30 minutes. After washing, the slides were incubated at 4°C overnight with rabbit polyclonal anti-Smad7 (Abcam, Cambridge, UK) diluted 1:100. The sections were incubated with FITC-labeled anti-rabbit goat IgG (Sigma-Aldrich, St. Louis, USA) diluted 1:50 for 1 h, and stained with 4,6-diamidino-2-phenylindole (DAPI, Thermo Scientific, Waltham, USA) for 7 min, and mounted in glycerol. Controls were treated by omitting the primary antibody. Sections were examined with a Nikon Eclipse 80i microscope (Tokyo, Japan). At least ten microscopic fields were taken from each rat under × 400 magnification using the 488 nm filter for Smad7 and 540 nm for DAPI. The ratio of Smad7 positive cells in relation to all cells present in the peritoneum (stained with DAPI) was calculated for every single picture, and the mean ratio was established for each rat.

### Multiplex for cytokines and MCP-1 chemokine

Peritoneal samples were lysed in RIPA buffer (EMD Millipore^R^, Billerica, USA) with protease inhibitor. A MILLIPLEX^R^ Map rat cytokine/chemokine assay kit (EMD Millipore^R^, Billerica, USA) was used to test for the concentration of TNF-α, IL-1β, monocyte chemoattractant protein-1 (MCP-1), and macrophage inflammatory protein-2 (MIP-2) in the peritoneal homogenate. The assay was read on the Bio-Plex suspension array system, and the data were analyzed using Bio-Plex Manager software version 4.0. Results were expressed as pg/mg protein.

### Quantitative real-time PCR

The mRNA expression of TGF-β, fibronectin, fibroblast specific protein-1 (FSP-1), Smad3, BMP-7, Smad7, TNF-α, IL-1β, and VEGF in peritoneal samples was analyzed using quantitative real time polymerase chain reaction (qPCR), as previously described [28]. Total RNA was extracted from peritoneal tissues by using the TRIZOL reagent (Ambion, Austin, TX). After total RNA reverse-transcribed to cDNA, qPCR was performed using the StepOne Plus Real-Time PCR system, and quantitative comparisons were obtained using the ΔΔCT method (Applied Biosystems, Singapore, Singapore). The primer sequences for amplifying the target genes are summarized in **S1 Table**. Primers for β-actin were used as an internal control.

### Capillary density

Immunofluorescence for Isolectin-B_4,_ an endothelial marker, was performed to study neoangiogenesis in the PM. Paraffin sections were deparaffinized and rehydrated by immersion in hot xylene 60°C followed by a graded series of ethanol. Antigen retrieval was performed by a heat-induced method with citrate buffer (pH 6.0) for 20 minutes, followed by incubation with blocking solution of 5% BSA in *PBS-Tween* (pH 7.4) at room temperature for 60 minutes. The slides were incubated at 4°C overnight with anti-isolectin B4 conjugated to Dy Ligth 594 *Griffonia Simplicifolia* (Vector Labs, Burlingame, US) diluted 1:100. The sections after washing were mounted with ProLong™ Gold anfifade with DAPI (Life Technologies, Eugene, USA). At least ten microscopic fields were taken from each rat under × 400 magnification using the 594 nm filter for isolectin and 540 nm for DAPI (Nikon Eclipse 80i microscope, Tokyo, Japan). For each animal, the number of Isolectin-B4 positive blood vessels in the PM were counted in all photomicrography fields. The density of capillaries in each slide was determined by the number of blood vessels divided by the area of PM and was expressed as number of vessels/mm^2^. A mean vascular index from each rat was calculated for statistical analysis.

### Statistical analysis

Data are presented as the mean ± standard error of mean (SEM) and statistical analyses were performed with the Prism statistical program (GraphPad, San Diego, USA). A one-way ANOVA with post hoc (Tukey) correction was used to compare the groups. A *p-*value < 0.05 was considered statistically significant.

## Results

### Overall outcome and body weight

No rat died during the experiment in any of the groups. The analysis of VPA administration in normal rats (Control+VPA group) demonstrated no effect of the drug on overall behavior and body weight (**S2 Table**). The PF group had a significantly lower weight gain compared with the Control group (*p*<0.01). Treatment of PF rats with VPA (PF+VPA group) recovered the normal body weight gain.

### VPA prevented experimental peritoneal fibrosis and ameliorated peritoneal transport function

**Fig 1:**
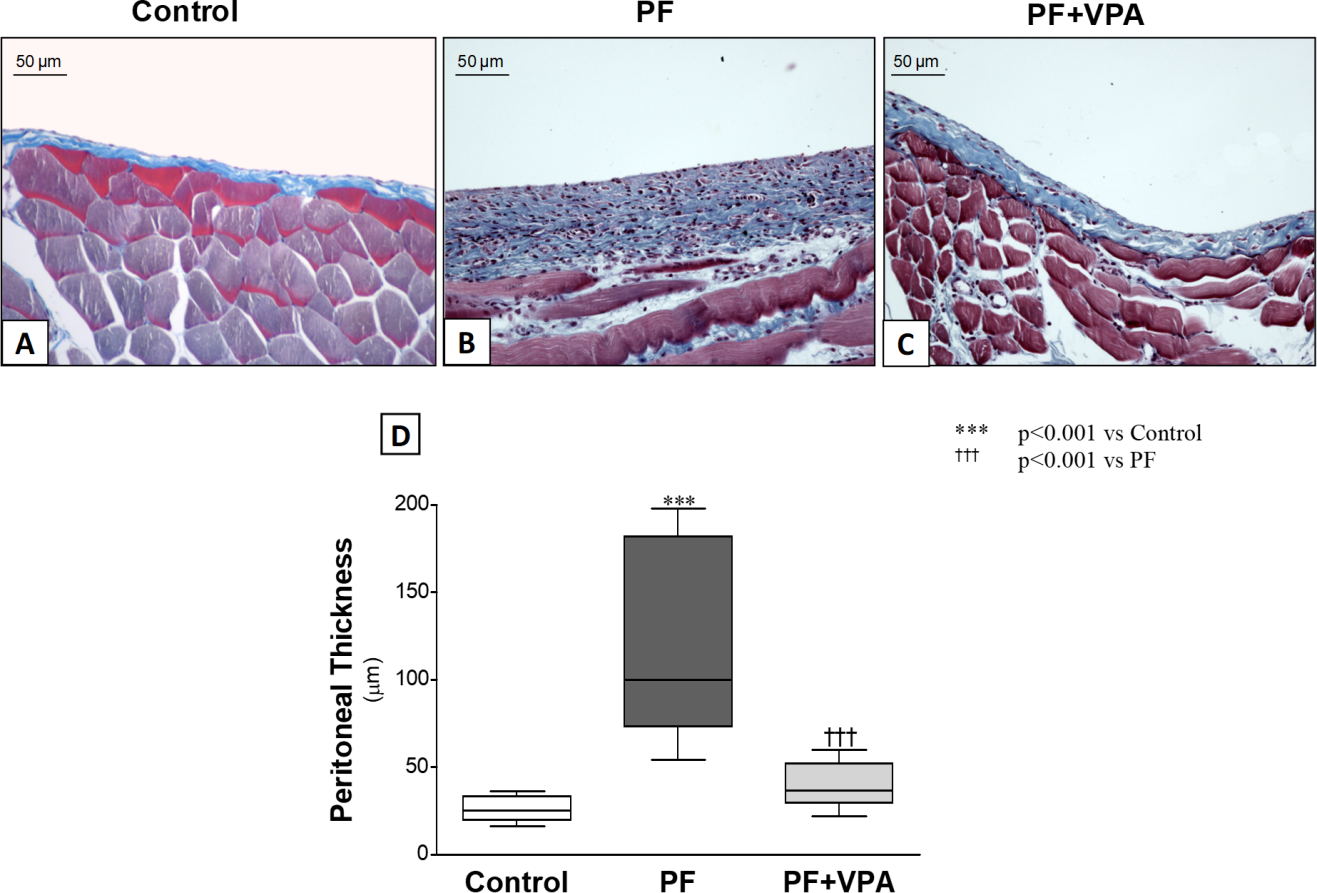
**Effect of VPA on the peritoneal thickness in experimental PF model (A-C):** Representative photomicrographs of peritoneal samples stained with Masson’s trichrome (x200)**. (A)** Control group (n = 8) showed a thin submesothelial layer of the peritoneum, without any morphological changes. **(B)** The peritoneal membrane in the PF group (n = 10) rats showed a marked thickening of the submesothelial compact zone. **(C):** VPA prevented the development of submesothelial thickening in the PF+VPA group (n = 10). **(D)** Quantification analysis of the effect of VPA on peritoneal thickness, at day 30.

As shown in **Fig 1**, the peritoneum of the control rats comprised a monolayer of mesothelial cells with a thin layer of connective tissue underneath. Otherwise, the peritoneal samples of the PF group had a marked thickening of the submesothelial compact zone (26.2 ± 3 vs 123.8 ± 18, respectively; *p<*0.001). In contrast, VPA treatment significantly reduced the peritoneal thickening (38.9 ± 5 μm; *p*<0.001). PM thickness was not affected by VPA as demonstrated in the in the Control+VPA group (21.0±5 μm; **S3 Table**).

To evaluate whether the PM morphological preservation induced by VPA had an impact on preserving peritoneal function, ultrafiltration (UF), glucose D_2_/D_0_ ratio (the D_2_/D_0_ (the ratio of 2-hour to 0-hour glucose concentration of the dialysate) to assess the transport of small solutes), and free water transport in the peritoneum (Na gap) were analyzed. Peritoneal function was significantly affected in the PF group demonstrated by UF, D_2_/D_0_, and Na gap values significantly lower than Control groups (*p*<0.001). VPA treatment preserved peritoneal function. Peritoneal function was not affected in the Control+VPA group (**Table 1**).

**Table 1:** Analysis of peritoneal membrane function.

	UF^1^ (mL)	D_2_/D_0_^2^	Na gap^3^ (meq/L)
**Control**	15.9 ± 3	0.18 ± 0.01	9.8 ± 0.7
**Control+VPA**^4^	15.2 ± 1	0.2 ± 0.01	10 ± 0.6
**PF**^5^	-1.0 ± 1***	0.11 ± 0.02***	1.8 ± 2.1**
**PF+VPA**	11.2 ± 2	0.21 ± 0.01	9.4 ± 0.5

Data are expressed as the mean ± SEM (n = 5/group).

^1^ultrafiltration volume

^2^the ratio of 2 hour to 0 hour glucose concentration of the dialysate

^3^Sodium gap, determined by subtracting the sodium concentration of drained volume from infused PD fluid

^4^Valproic acid

^5^Peritoneal fibrosis.

**p<0.01

***p<0.001 compared with Control group and VPA group.

### VPA ameliorated peritoneal fibrosis by attenuating the expression of fibrotic markers and by decreasing the number of myofibroblasts in the peritoneum

**Fig 2:**
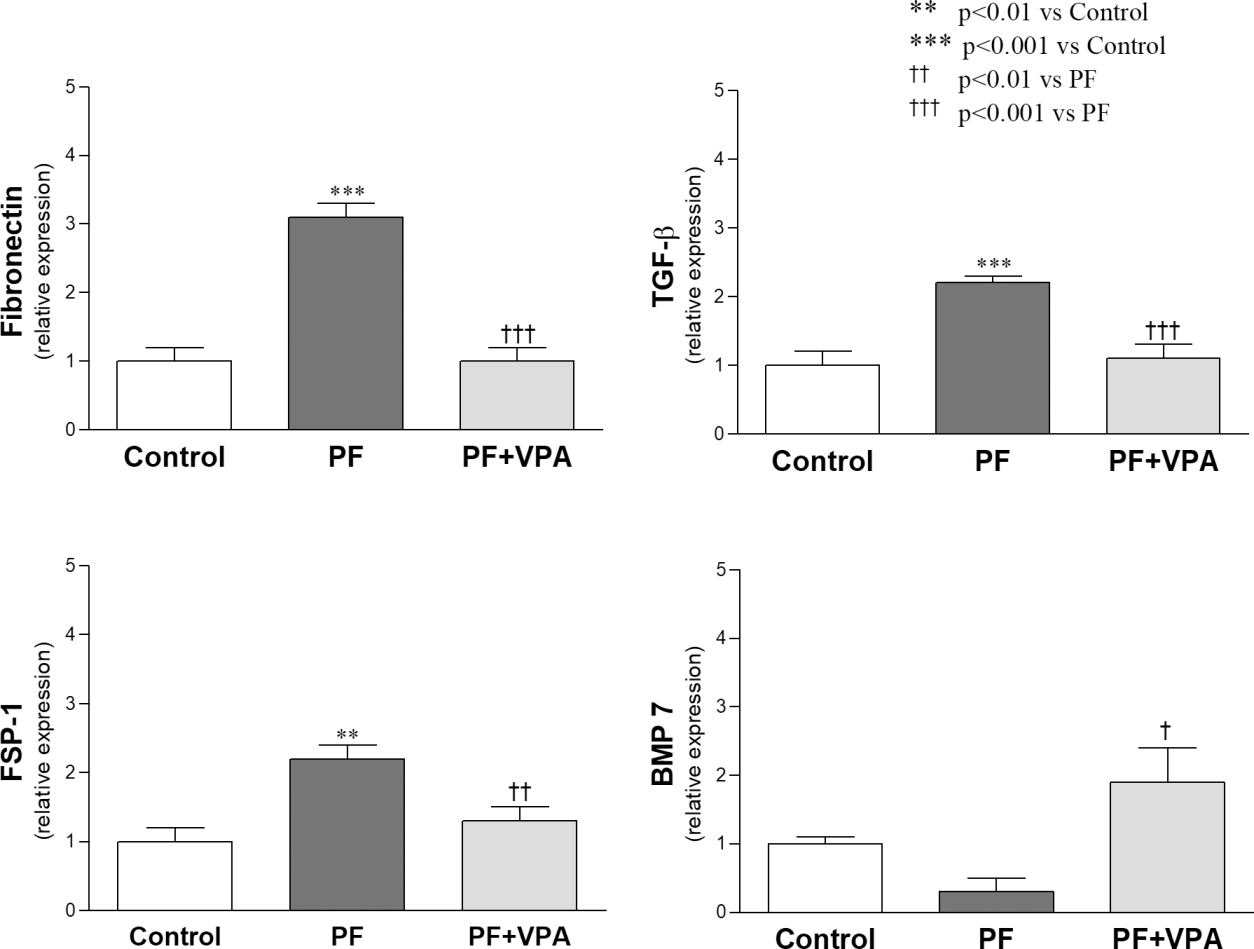
Expression of fibronectin, TGF-β, FSP-1, and BMP-7 genes in the peritoneal tissue by qPCR. VPA treatment reduced the upregulation of the profibrotic genes observed in the PF group and increased BMP-7 expression depressed in the PF group (n = 6/group).

The expression of fibrotic markers, such as fibronectin, TGF-ß and fibroblast specific protein-1 were analyzed in the peritoneum by means of qPCR. The mRNA expression of these genes was significantly increased in the PF group when compared with normal controls (*p*<0.001, **S4 Table**). The VPA treatment significantly reduced them to control levels (**Fig 2**). On the other hand, we found that the expression of BMP-7, an antifibrotic factor that counteracts TGF-ß action, was significantly higher in the PF+VPA group compared with the PF group (*p*<0.05; **Fig 2**, **S4 Table**).

**Fig 3:**
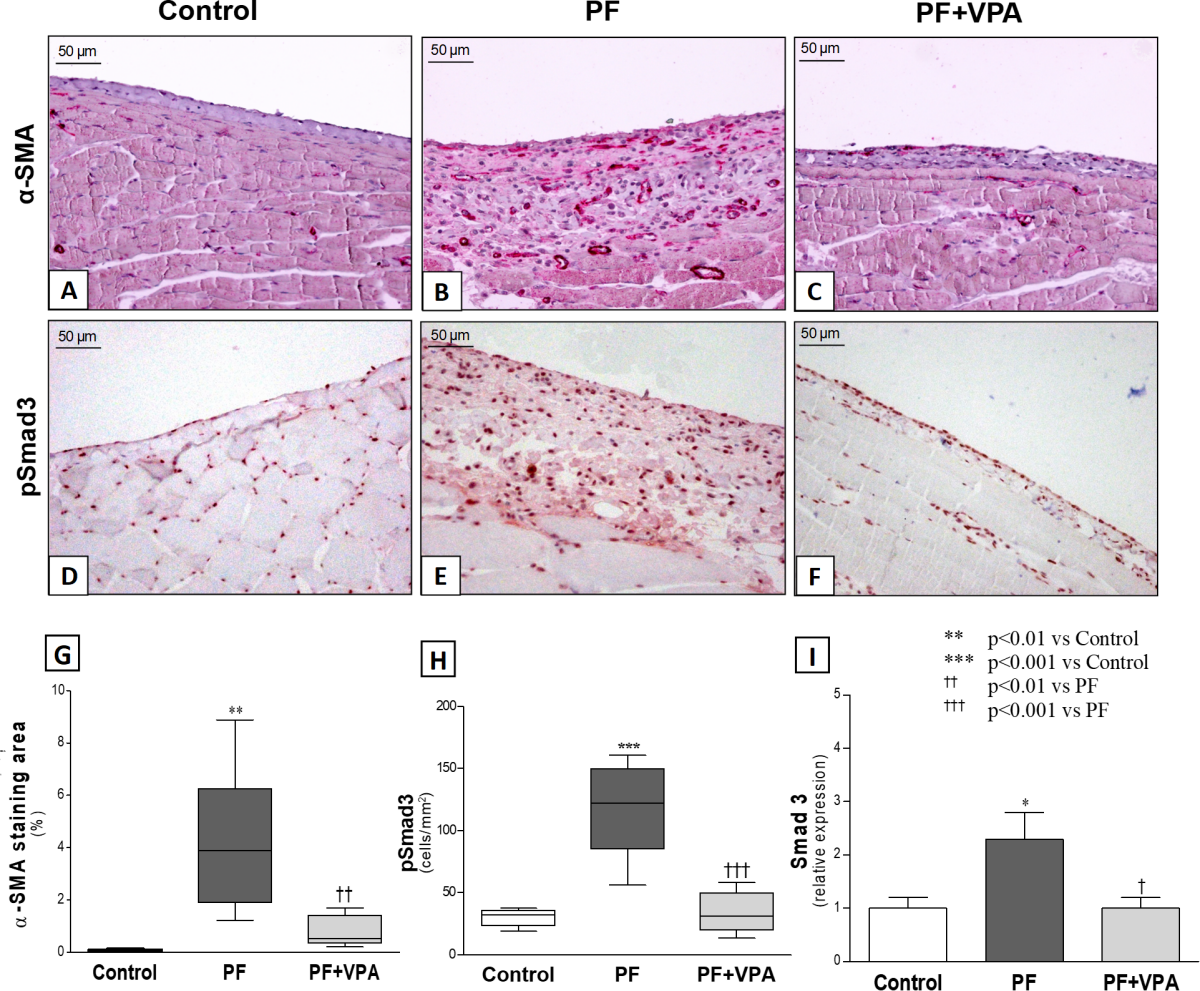
Representative photomicrographs of parameters related to experimental peritoneal fibrosis and effect of treatment with VPA (x200). **(A-C)** Effect of VPA on the peritoneal expression of α-SMA (myofibroblasts) by immunohistochemistry. **(A):** No α-SMA expression was detected in the peritoneal membrane of the Control group. **(B)** Induction of PF was associated with a marked increase in α-SMA expression. **(C)** A striking reduction of myofibroblasts was observed with VPA treatment. **(D-F)** Effect of VPA on the peritoneal expression of pSmad3, by immunohistochemistry. **(D)** Only a few positive cells were noted in the peritoneal membrane of the Control group; **(E)** PF group showed an increased number of pSmad3 positive cells (stained in brown) infiltrating peritoneal membrane; **(F)** pSmad3 expression was significantly reduced by VPA treatment. **(G-H)** Quantification analysis of the effect of VPA in experimental PF, regarding α-SMA staining area, and the number of pSmad3 positive cells in all groups (n = 5-6/group). **(I)** Quantification analysis of the effect of VPA in the peritoneal membrane expression of mRNA Smad3 in all groups (n = 6/group).

Moreover, we evaluated the expression of α-SMA, which is a marker of myofibroblasts, by immunohistochemistry (**Fig 3**). In the Control group, very few positive α-SMA cells were observed in the peritoneum. The VPA treatment reduced the α-SMA staining area from 4.2±0.4% in the PF group to 0.8 ± 0.1% in the PF+VPA group (*p*<0.001; **S5 Table**).

### VPA protected against PF, possibly by interfering in the TGF-β/Smad pathway

Smad3 and Smad7, which are critical intracellular mediators involved in TGF-β signaling, were also analyzed. While intracellular phosphorylation of Smad3 leads to the transcription of profibrotic genes, Smad7 upregulation has been recognized as a critical inhibitor of TGFβ/Smad signaling. The peritoneal expression of pSmad3 (**Fig 3H**) and Smad3 mRNA (**Fig 3I**) was significantly higher in the PF groups compared with the Control group. However, treatment with VPA significantly reduced the number of pSmad3^+^ cells and its gene expression in the PM (**Fig 3, S5 Table**).

**Fig 4:**
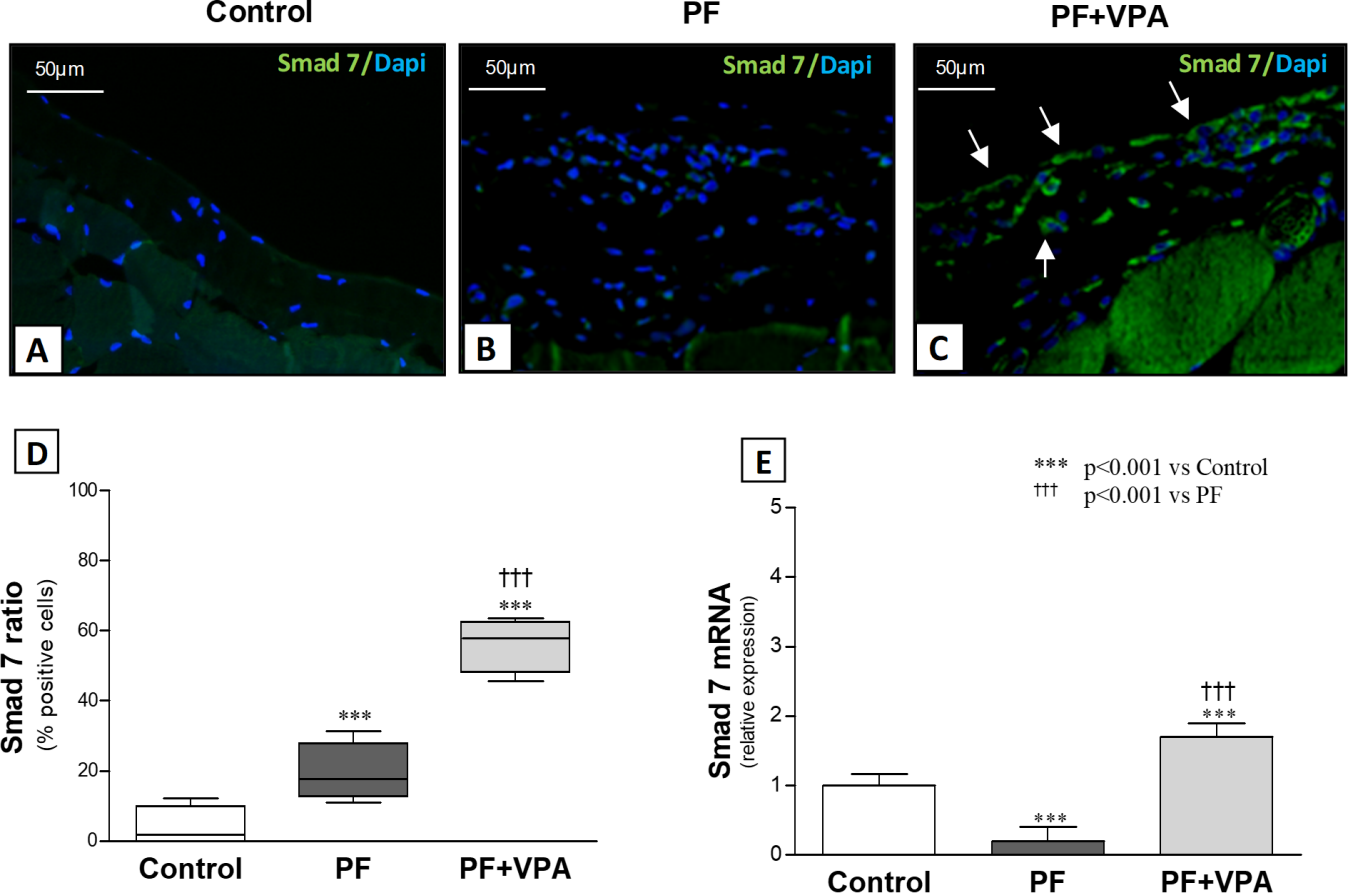
Smad7 expression in the peritoneal membrane. **(A-C)** Representative immunofluorescence photomicrographs of Smad7^+^ cells (stained in green) and DAPI^+^ cells (stained in blue) in the peritoneal membrane of the different groups (x400). Almost no Smad7 expression was detected in the Control group (n = 4), and only few Smad7+ cells were observed in the PF group (n = 5); In the PF+VPA group (n = 4) most of cells in the peritoneal membrane were stained for Smad7 (white arrows). **(D)** Quantification analysis demonstrated Smad7+ cells ratio to all cells of peritoneal membrane; **(E)** Effect of VPA on the expression of Smad7 mRNA (n = 6/group), by qPCR.

On the other hand, PF animals treated with VPA showed an upregulated expression of Smad7, detected by immunofluorescence and qPCR (**Fig 4**). In the control group and in the PF group only few Smad7^+^ cells were detected (3.9 ± 2.8% and 19.8 ± 3.6%, respectively). However, the ratio of Smad7^+^ cells to all cells in the peritoneal membrane of PF rats treated with was significantly increased (56.2 ± 3.4%; *p*<0.001; **Fig 4D**). In parallel, Smad7 mRNA levels were reduced in the PF group, but significantly upregulated in the PF+VPA group (**Fig 4E**, **S6 Table**).

### VPA reduced inflammation and neoangiogenesis in the PF experimental model

**Fig 5:**
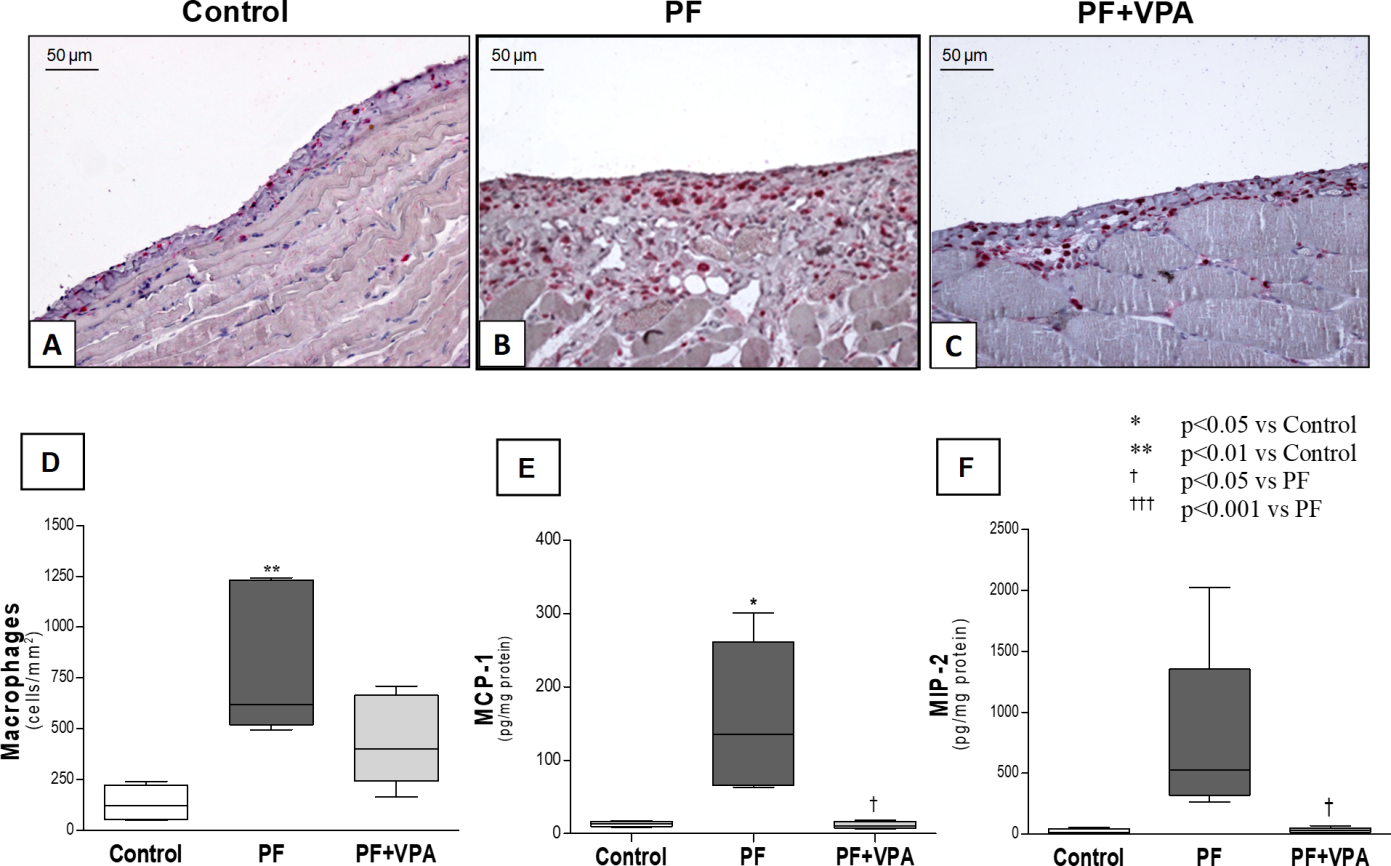
Peritoneal membrane infiltration of macrophages and expression of macrophage-related chemokines. **(A-C)** Representative photomicrographs of immunohistochemistry for macrophages (ED1^+^) (x200). **(A):** Only few ED1^+^ cells were present in the normal peritoneal membrane; **(B)** PF group showed a marked increase in the number of macrophages infiltrating peritoneal membrane; **(C)** Animals with PF treated with VPA exhibited a non-significant reduction of macrophage infiltration. **(D)** Quantitative analysis of the number of macrophages of all groups (n = 5/group). **(E-F)** Concentration of MCP-1 and MIP-2 in the peritoneal tissue, detected by Multiplex. VPA treatment reduced the protein levels of macrophage chemoattractant proteins (n = 4-5/group).

**Fig 6:**
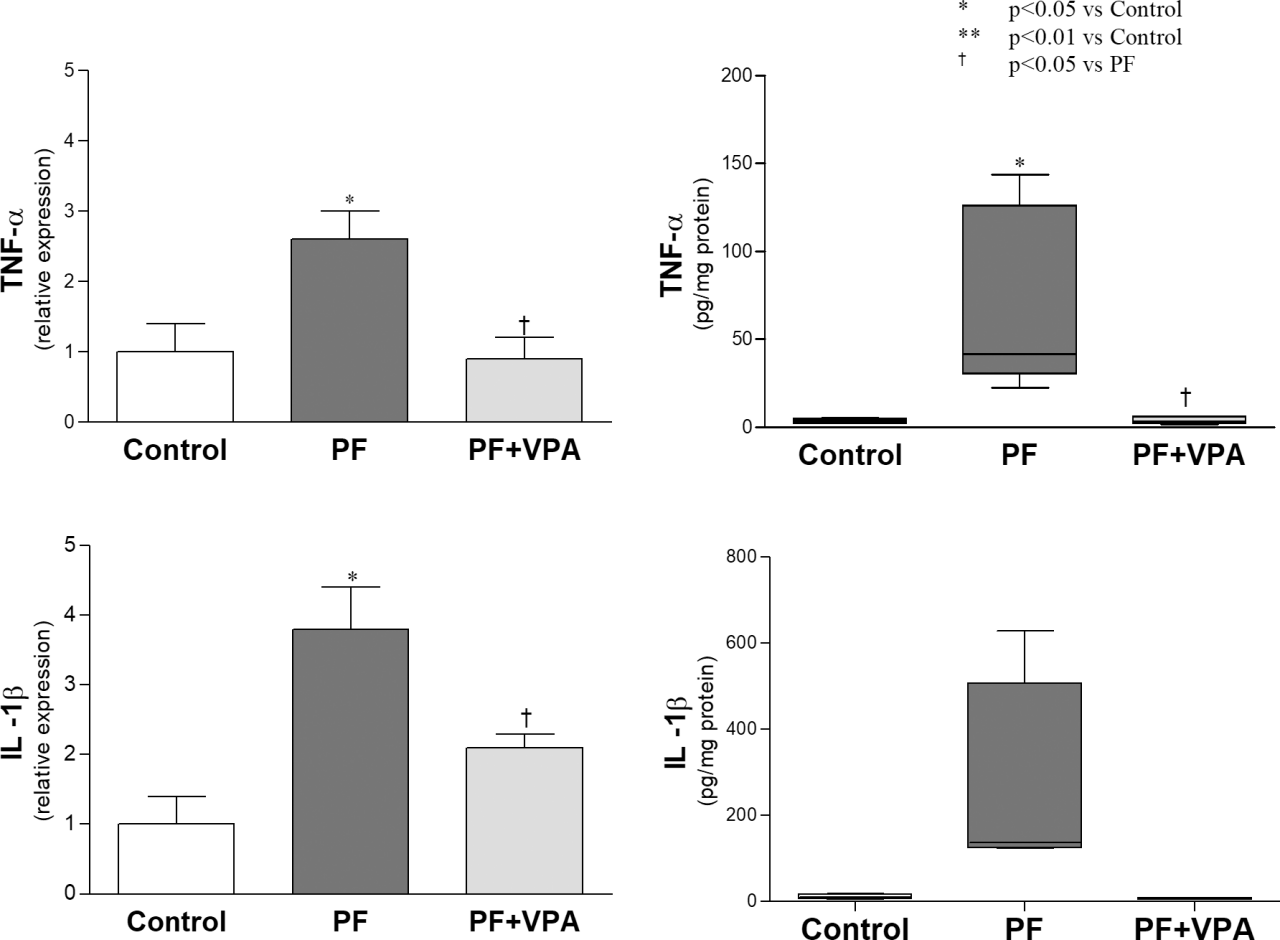
Expression of proinflammatory cytokines in the peritoneal membrane of the different groups. VPA treated animals showed a significant lower gene (n = 6/group) and protein expression (n = 4-5/group) of TNF-α in peritoneal tissue than the PF group.

To examine possible anti-inflammatory effects of VPA on the peritoneal membrane, macrophages and the proinflammatory cytokines and chemokines were analyzed by immunohistochemistry, qPCR and Multiplex. In the peritoneum of the PF group, the number of macrophages (ED1^+^ cells) and the concentration of macrophage-chemoattractants (MCP-1, Monocyte chemoattractant protein-1, and MIP-2, Macrophage inflammatory protein) were increased compared with the Control group (**Fig 5**). No significant reduction in the number of macrophages in the peritoneal membrane of animals with PF treated with VPA was observed (**Fig 5D**; **S5 Table**). However, the increased levels of MCP-1 and MIP-2 observed in the PF group were reduced by VPA administration (**Fig 5E and 5F; S7 Table**). Similarly, VPA-treated animals showed a significantly lower gene and protein expression of TNF-α in peritoneal tissue when compared with PF group (**Fig 6, S7 and S8 Tables).**

**Fig 7:**
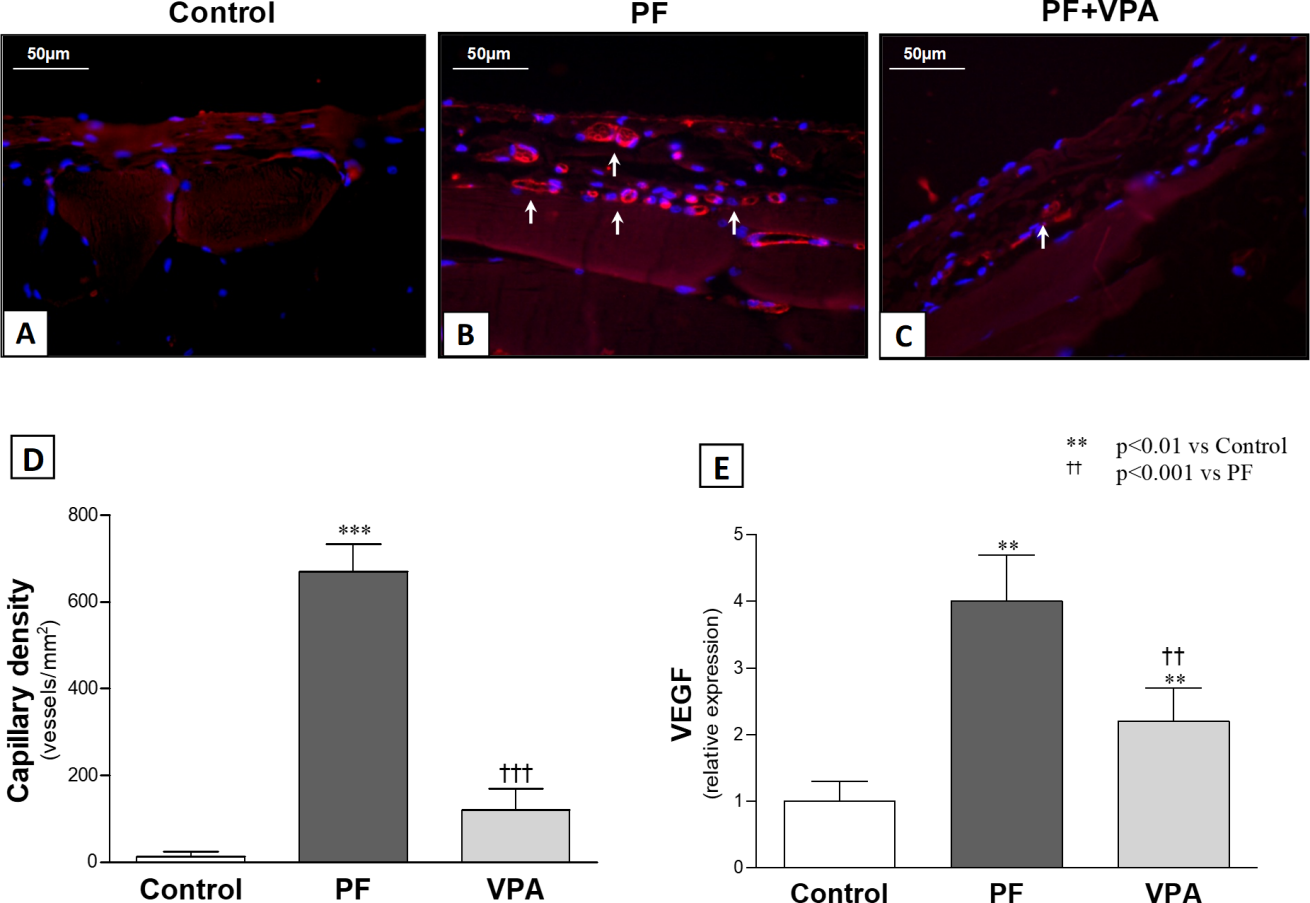
Capillary density and VEGF mRNA expression in the peritoneum. **(A-C)** Representative immunofluorescence photomicrographs of Isolectin-B_4_ (stained in red), an endothelium marker, and DAPI+ cells (stained in blue) in the peritoneal membrane. While virtually no vessels were identified in Control Group **(A),** an intense neoangiogenesis (white arrows) in the submesothelial zone was observed in the PF group **(B)**. **(C)** VPA treatment attenuated this increase. **(D)** Graph comparing the vascular density of all groups(n = 3-4/group). **(E)** Expression of mRNA VEGF in peritoneal tissue by PCR (n = 6/group).

VPA also demonstrated antiangiogenic effects. The capillary density in the PM was significantly increased in the PF group compared with the Control group. The PF+VPA group showed a significant reduction of the number of peritoneal vessels compared with PF group (**Fig 7**). The results for the gene expression of angiogenic factor VEGF paralleled the capillary density findings (**Fig 7E**).

## Discussion

In this study, the administration of VPA prevented the progression of peritoneal fibrosis induced by GC in rats. VPA treatment also reduced myofibroblast infiltration and attenuated the upregulated expression of profibrotic and proinflammatory genes observed in untreated animals, in addition to preserving peritoneal ultrafiltration capacity. These findings are consistent with previous reports that studied the antifibrotic effect of VPA and others HDAC inhibitors [12, 18, 20]. VPA is an attractive HDACi because it has been clinically used for decades to treat epilepsy and it is readily available on the market.

Recently, it was shown that treatment of diabetic rats with VPA 300 mg/kg by oral route for 8 consecutive weeks promoted histone acetylation, ameliorated fibrosis, and decreased the expression of TGF-β1, α-SMA and fibronectin in the kidney [24]. Moreover, Io *et al* demonstrated the protective effect of suberoylanilide hydroxamic acid (SAHA), a class I HDAC inhibitor similar to VPA, on the progression of peritoneal fibrosis in mice[14]. Although previous studies highlighted that VPA and other HDAC inhibitors are potent antifibrotic drugs [13, 24, 29, 30], their underlying mechanisms remain unknown. To our knowledge, this is the first study to investigate the effect of VPA on peritoneal fibrosis.

The fibrous thickening of the PM and the expression of α-SMA, a myofibroblast marker, were reduced by VPA treatment in comparison with the PF group. VPA administration also blocked the upregulation of profibrotic factors, notably, TGF-β and fibronectin. Van Beneden *et al* demonstrated that VPA antifibrotic effects in the adriamicin nephropathy model were associated with reduced expression of genes related to fibrosis development such as TGF-β and α-SMA [12, 18]. Furthermore, the HDACi Tricostatin A suppressed epithelial-to-mesenchymal transition (EMT) induced by TGF-β in human renal epithelial cells culture [31]. Although the role of EMT in peritoneal fibrosis had been recently challenged [32], the inhibitory effect of VPA on the EMT, demonstrated by the reduction of α-SMA expression, may account in part for its antifibrotic effect. Furthermore, the administration of VPA induced a higher mRNA expression of the antifibrotic factor BMP-7 in the PF+VPA group compared with PF group. BMP-7 and TGF-β shares similar downstream Smad signaling pathways, but counter regulating each other. BMP-7 signals directly antagonize TGF-β1 signals inhibiting fibrosis [33]. Similar to our findings, Io and colleagues demonstrated that the HDACi SAHA induced a higher expression of BMP-7 and prevented the peritoneal fibrosis induced by GC mice [14].

The TGF/Smad signaling pathway plays a pivotal role in peritoneal fibrosis. The Smad3 phosphorylation is one of the hallmarks of activation of the TGF-β receptors [34]. Duan and colleagues showed that Smad3 knockout mice were protected from PF development induced by long-term exposure to PD fluids [35]. In our study, the blockage of TGF-β/Smad pathway, demonstrated by lower levels of Smad3 mRNA and Smad3 phosphorylated positive cells in the peritoneum of the treated animals, contributed significantly to antifibrotic effect of VPA. On the other hand, an overexpression of Smad7 has been shown to antagonize TGF-β mediated fibrosis and inflammation, suggesting a therapeutic potential of Smad7 to treat these conditions [9]. Guo *et al*. demonstrated that in rats transfected with a Smad7 transgene, peritoneal fibrosis induced by PD was attenuated and Smad2/3 activation was inhibited [9]. In our study, the treatment with VPA induced a higher expression of the Smad7 gene in comparison to PF group. Additionally, a significant increase in the number of Smad7 positive cells in the peritoneum of PF+VPA group rats was detected by immunofluorescence. The degradation of Smad7 is regulated by the balance between acetylation, deacetylation, and ubiquitination. The acetylation of Smad7 inhibits its ubiquitination and proteasome-mediated degradation [36]. Smad7 interact with HDACs and is deacetylated by these enzymes [16, 17]. VPA could have enhanced Smad7 expression through post-transcriptional mechanisms by inhibiting Smad7 deacetylation and degradation.

There is increasing evidence on the anti-inflammatory effects of VPA [19]. A trend toward the reduction in the number of MΦ in the PM accompanied by a significant reduction of MΦ chemokines (MCP-1 and MIP-2) was found in the PF+VPA group compared with PF group. Through unknown mechanisms, VPA was shown to reduce the expression of MCP-1 and MΦ infiltration in different experimental models [12, 37]. Thus, the inhibition of MCP-1 and MIP-2 expression by VPA may have attenuated the macrophage infiltration and inflammation of PM. Additionally, in our experiments, VPA treatment reduced significantly the upregulation of proinflammatory cytokines (TNF-α and IL-1β) induced by CG in the peritoneum. A previous study showed that VPA significantly repressed the production of TNF-α in the mouse macrophage cell line RAW264.7 stimulated with LPS [38]. Therefore, the inhibition of TNF-α production by VPA may contribute to its anti-inflammatory effect.

In the PF group, CG induced a marked increase in peritoneal vascularization and VEGF mRNA expression that was attenuated by VPA treatment. These findings may be due to the anti-inflammatory effect of VPA, but a direct effect of VPA in the VEGF regulation is possible. For example, VPA inhibited mRNA and protein expression of VEGF and VEGFR2 in leukemic cells transplanted into mice preventing angiogenesis and tumor growth. Since peritoneal fibrosis and angiogenesis are closely related in the response of the peritoneum to continuous injury[39], target therapies that reduce VEGF expression are promising.

Long-term exposure to nonphysiologic PD fluids solutions leads to neoangiogenesis with ensuing UF failure resulting from an increase in peritoneal membrane vascular surface area, faster peritoneal solute transport rate, and early dissipation of the osmotic gradient[40]. Changes in the glucose transport ratio (D_t_\D_0_), have been used to monitor solute transport ratio and PM function over time. In our study, PF group showed a lower D_2_\D_0_ value, indicating a faster peritoneal absorption of glucose. As expected, since VPA reduced neoangiogenesis in the PM, it prevented an increase in glucose transport rate induced by GC. In addition to capillary surface area, the diffusion length between the dialysate and the mesothelium also plays an important role in the transport characteristics of the peritoneum. An increase in collagen density in the peritoneal interstitium have been suggested to constitute a second barrier outside the capillaries, restricting osmotic free water transport without affecting small solute transport [40]. The sodium sieving, that is, the fall in dialysate sodium concentration during the first hours of a dwell with hypertonic glucose, is used to monitor free water transport in PD patients. The absence of the initial drop of sodium in dialysate, which is a surrogate marker of reduced osmotic conductance of the PM, has been suggested as an early predictor of EPS [41]. In our study, different from PF+VPA and Control groups, we found a reduction in the sodium dialysate drop (Na Gap) in the PF group. Therefore, the loss of UF capacity loss in the PF group could be explained by an increase in glucose transport rate secondary to hypervascularization of PM as well as a resistance to free water transport imposed by peritoneal fibrosis.

Our study has some limitations for clinical application. The administration of VPA and CG in the present study were started simultaneously. Thus, the effect of VPA initiation subsequent to an established peritoneal fibrosis is unknown. Additionally, a dose-response study to determine the lowest effective dose of VPA in the treatment of peritoneal fibrosis was not done, and the effective and safe dose of VPA for rats may be different from that used for humans. Further studies are needed to clarify whether VPA can be used to reduce peritoneal fibrosis in PD patients.

In conclusion, we have shown that VPA inhibits the progression of peritoneal fibrosis in a CG-induced peritoneal fibrosis model in rats. VPA inhibited different and important mechanisms involved in peritoneal membrane modifications induced by PD, as activation of TGF-β/Smad pathway, inflammation, and angiogenesis. Notably, VPA induced the expression of antifibrotic factors BMP-7 and Smad7. Our results are interesting and shed light on a new perspective for the treatment of peritoneal fibrosis. However, this is an exploratory study and future studies are needed before translating this experimental finding into clinical application.

## Supporting information

S1 Table Primer sequences used for qPCR. (DOCX)

S2 Table Average body weight in the different groups. (DOCX)

S3 Table Peritoneal membrane thickness. (DOCX)

S4 Table mRNA relative expression to control for pro-fibrotic genes and BMP-7. (DOCX)

S5 Table Average expression of myofibroblasts (α-SMA), macrophages number (ED1+), and phosphorylated Smad 3 positive cells (phospho-Smad3) in the peritoneal membrane by immunohistochemistry. (DOCX)

S6 Table mRNA relative expression to control for Smad7. (DOCX)

S7 Table Multiplex analyses of inflammatory cytokines/chemokines concentration in the peritoneal tissue. (DOCX)

S8 Table mRNA relative expression to control for proinflammatory genes. (DOCX)
