# Supplementary material for: Anti-fibrotic effects of valproic acid in experimental peritoneal fibrosis
Source: PLoS One. 2017 Sep 5;12(9):e0184302. doi: 10.1371/journal.pone.0184302 (PMC5584960; doi:10.1371/journal.pone.0184302)
Supplement: S1 Table — (DOCX) [file pone.0184302.s001.docx]

**S1 Table.** **Primer sequences used for qPCR.**

| **Primer** | **Sequence of PCR primers** |
| --- | --- |
| **IL-1β** | Forward 5’-CCTTGTGCAAGTGTCTGAAGCAGC-3’  Reverse 5’-GCCACAGCTTCTCCACAGCCA-3’ |
| **TNF- α** | Forward 5’-ATCTGAGGGCTCGCCCGGT-3’  Reverse 5’-CAATGGCAGCACCGCCACCA-3’ |
| **VEGF** | Forward 5’-ACTGTGAGCCTTGTTCAGAGCGG3’  Reverse 5’-TCAAGCTGCCTCGCCTTGCA3’ |
| **TGF-β** | Forward 5’-CAACCCGGGTGCTTCCGCAT-3’  Reverse 5’-TGCTCCACCTTGGGCTTGCG -3’ |
| **Fibronectin** | Forward 5’-TGACCCAGACTTACGGTGGCA-3’  Reverse 5’-GGAGTAGAAGGTCCTACCGTTGTAGTG-3’ |
| **FSP-1** | Forward 5’-GGCAACGAGGGTGACAAGTT-3’  Reverse 5’-CCCTGGTCAGTAGTCCCTTGA-3’ |
| **Smad3** | Forward 5’-TCAACGGAACTTGGGAATGAG-3’  Reverse 5’-TCACCTCGATCTTGACCTTTTGT-3’ |
| **BMP-7** | Forward 5’-TGGCGGTGGAGGAGAGTGGG-3’ Reverse 5’-CTGCAGGCTGGCCAAAGGGG-3’ |
| **Smad7** | Forward 5’-GCGGATCCCTTGGAAAGG-3’  Reverse 5’-GCCACAGCTTCTCCACAGCCA-3’ |
| **Β-actin** | Forward 5’-AGGAGTACGATGAGTCCGGCCC-3’  Reverse 5’-GTAGTGCGGAGCTCTCCTTCA-3’ |
