# Supplementary material for: Anti-fibrotic effects of valproic acid in experimental peritoneal fibrosis
Source: PLoS One. 2017 Sep 5;12(9):e0184302. doi: 10.1371/journal.pone.0184302 (PMC5584960; doi:10.1371/journal.pone.0184302)
Supplement: S2 Table — (DOCX) [file pone.0184302.s002.docx]

**S2 Table. Average body weight in the different groups.**

|  | **Day 0**  (g) | **Day 30**  (g) | **Δ**  (g) |
| --- | --- | --- | --- |
| **Control** | 296 ± 4 | 392 ± 11 | 96 ± 5 |
| **Control+VPA** | 292 ± 3 | 384 ± 2 | 91 ± 3 |
| **PF** | 291 ± 7 | 347 ± 11^*^ | 56 ± 8^**^ |
| **PF+VPA** | 295 ± 7 | 376 ± 14 | 81 ± 7 |

Data are expressed as the mean ± SEM. ^*^p<0.05, ^**^p<0.01. Δ = weight gain throughout experiment.
