# Supplementary material for: Anti-fibrotic effects of valproic acid in experimental peritoneal fibrosis
Source: PLoS One. 2017 Sep 5;12(9):e0184302. doi: 10.1371/journal.pone.0184302 (PMC5584960; doi:10.1371/journal.pone.0184302)
Supplement: S3 Table — (DOCX) [file pone.0184302.s003.docx]

**S3 Table. Peritoneal membrane thickness.**

|  | **Peritoneal thickness**  (µm) |
| --- | --- |
| **Control** | 26.2 ± 3 |
| **Control+VPA** | 21.0 ± 5 |
| **PF** | 123.8 ± 18^***^ |
| **PF+VPA** | 38.9 ± 5^†††^ |

Data are expressed as the mean ± SEM. PF = peritoneal fibrosis; VPA = valproic acid;
 ^***^p<0.001 compared with the Control group; ^†††^p<0.001 compared with PF group.
