# Supplementary material for: Anti-fibrotic effects of valproic acid in experimental peritoneal fibrosis
Source: PLoS One. 2017 Sep 5;12(9):e0184302. doi: 10.1371/journal.pone.0184302 (PMC5584960; doi:10.1371/journal.pone.0184302)
Supplement: S4 Table — (DOCX) [file pone.0184302.s004.docx]

**S4 Table. mRNA relative expression to control for pro-fibrotic genes and BMP-7.**

|  | **TGF-β** | **Fibronectin** | **FSP-1** | | **BMP-7** |
| --- | --- | --- | --- | --- | --- |
| **Control** | 1 ± 0.2 | 1 ± 0.2 | | 1 ± 0.2 | 1.0 ± 0.1 |
| **PF** | 2.2 ± 0.1^***^ | 3.1 ± 0.2^***^ | | 2.2 ± 0.1^**^ | 0.3 ± 0.5 |
| **PF+VPA** | 1.1 ± 0.2^†††^ | 1.0 ± 0.2^†††^ | | 1.3 ± 0.2^††^ | 1.0 ± 0.2^†^ |

Data are expressed as the mean ± SEM. TGF = transforming growth factor; FSP-1 = fibroblast specific protein, BMP = Bone Morphogenic Protein; PF = peritoneal fibrosis; VPA = valproic acid. ^*^p<0.05, ^**^p<0.01, ^***^p<0.001 compared with Control group; ^†^p<0.05, ^††^p<0.01, ^†††^p<0.001 compared with PF group.
