# Supplementary material for: Anti-fibrotic effects of valproic acid in experimental peritoneal fibrosis
Source: PLoS One. 2017 Sep 5;12(9):e0184302. doi: 10.1371/journal.pone.0184302 (PMC5584960; doi:10.1371/journal.pone.0184302)
Supplement: S5 Table — (DOCX) [file pone.0184302.s005.docx]

**S5 Table. Average expression of myofibroblasts (α-SMA), macrophages number (ED1+), and phosphorylated Smad 3 positive cells (phospho-Smad3) in the peritoneal membrane by immunohistochemistry.**

|  | | **α-SMA**  (% area) | | **ED1^+^**  (cells/mm^2^) | | **p-Smad3**  (cells/mm^2^) | |
| --- | --- | --- | --- | --- | --- | --- | --- |
| **Control** | 0 ± 0 | | 133 ± 90 | | 30.1 ± 5 | |  |
| **PF** | 4.2 ± 0.4^**^ | | 826 ± 375^**^ | | 118 ± 17^***^ | |  |
| **PF+VPA** | 0.8 ± 0.1^††^ | | 443 ± 222 | | 34 ± 7^†††^ | |  |

Data are expressed as the mean ± SEM. PF = peritoneal fibrosis; VPA = valproic acid;
α-SMA = smooth muscle actin. ^**^p<0.01, ^***^p<0.001 compared with the Control group; ^††^p<0.01, ^†††^p<0.001 compared with PF group.
