# Supplementary material for: Anti-fibrotic effects of valproic acid in experimental peritoneal fibrosis
Source: PLoS One. 2017 Sep 5;12(9):e0184302. doi: 10.1371/journal.pone.0184302 (PMC5584960; doi:10.1371/journal.pone.0184302)
Supplement: S6 Table — (DOCX) [file pone.0184302.s006.docx]

**S6 Table. mRNA relative expression to control for Smad7.**

|  | **Smad7** |
| --- | --- |
| **Control** | 1.0 ± 0.1 |
| **PF** | 0.2 ± 0.2^***^ |
| **PF+VPA** | 1.7 ± 0.2^*** †††^ |

Data are expressed as the mean ± SEM. PF = peritoneal fibrosis; VPA = valproic acid. ^***^p<0.001 compared with Control group; ^†††^p<0.01 compared with PF group.
