# Supplementary material for: Anti-fibrotic effects of valproic acid in experimental peritoneal fibrosis
Source: PLoS One. 2017 Sep 5;12(9):e0184302. doi: 10.1371/journal.pone.0184302 (PMC5584960; doi:10.1371/journal.pone.0184302)
Supplement: S7 Table — (DOCX) [file pone.0184302.s007.docx]

**S7 Table. Multiplex analyses of inflammatory cytokines/chemokines concentration in the peritoneal tissue.**

|  | **MCP-1**  **(pg/mg)** | **MIP-2**  **(pg/mg)** | **TNF-α**  **(pg/mg)** | | **IL1-β**  **(pg/mg)** |
| --- | --- | --- | --- | --- | --- |
| **Control** | 13.5 ± 2.5 | 23 ± 10 | | 3.8 ± 0.6 | 10.4 ± 3 |
| **PF** | 158 ± 45^*^ | 774 ± 319 | | 71 ± 23^*^ | 255.5 ± 124 |
| **PF+VPA** | 11 ± 2.5^†^ | 31 ± 10^†^ | | 3.9 ± 0.8^†^ | 6.5 ± 0.7 |

Data are expressed as the mean ± SEM. MCP-1 = Monocyte chemoattractant protein; MIP-2 = macrophage inflammatory protein; PF =peritoneal fibrosis; VPA = valproic acid; TNF = tumoral necrosis factor. ^*^p<0.05 compared with Control group; ^†^p<0.05 compared with PF group.
