# Supplementary material for: Anti-fibrotic effects of valproic acid in experimental peritoneal fibrosis
Source: PLoS One. 2017 Sep 5;12(9):e0184302. doi: 10.1371/journal.pone.0184302 (PMC5584960; doi:10.1371/journal.pone.0184302)
Supplement: S8 Table — (DOCX) [file pone.0184302.s008.docx]

**S8 Table. mRNA relative expression to control for proinflammatory genes.**

|  | **TNF-α** | **IL-1β** |  |
| --- | --- | --- | --- |
| **Control** | 1 ± 0.4 | 1 ± 0.4 | |
| **PF** | 2.6 ± 0.4^*^ | 3.8 ± 0.6^*^ | |
| **PF+VPA** | 0.9 ± 0.3^†^ | 2.1 ± 0.2^†^ | |

Data are expressed as the mean ± SEM. PF = peritoneal fibrosis; VPA = valproic acid; TNF = tumoral necrosis factor. ^*^p<0.05, ^**^p<0.01 compared with Control group; ^†^p<0.05, ^††^p<0.01 compared with PF group.
